# DSSylation, a novel guide for protein degradation?

**DOI:** 10.1007/s13238-014-0020-9

**Published:** 2014-01-29

**Authors:** Cheng-Xin Gong

**Affiliations:** Department of Neurochemistry, Inge Grundke-Iqbal Research Floor, New York State Institute for Basic Research in Developmental Disabilities, Staten Island, NY 10314 USA

The natural design for the generation and the fate of proteins in an organism could be as complicated as life itself. A protein is biosynthesized strictly according to the message carried by the mRNA transcribed from its gene. However, when, where, what and how much a gene is expressed are regulated precisely and dynamically at multiple levels, including at transcription, mRNA maturation, alternative splicing, and translation levels. A newly synthesized protein often needs to be further modified and sorted posttranslationally for its optimal confirmation and function. When a protein reaches its life span, the senescent protein is degraded by protein degradation machinery involving the ubiquitin-proteosome system and lysosomes. Protein homeostasis is essential for maintaining the normal morphology and function of the cell.

There are many different posttranslational modifications of proteins, such as phosphorylation, acylation, methylation, glycosylation, glycation, truncation, ubiquitination, and oxidation. Some of these modifications are crucial for the maturation, proper folding, and biological activity of a protein, while others label a protein for degradation. In this issue of Protein & Cell, Zhang et al. (2014) reported a novel posttranslational modification of protein, named DSSylation, by which a protein is conjugated with DSS1, a small and highly acidic and conserved protein encoded by the gene *DSS1* (deleted in split hand/split foot 1). The lack of this gene is found in patients with a dominantly inherited heterogeneous limb developmental disorder called ectrodactyly or split hand/split foot malformation type 1 (Crackower et al., 1996).

Zhang et al. demonstrated both *in vitro* and in cultured cells that DSS1 forms SDS-resistant adducts with cellular proteins. The formation of DSS1-protein adducts is dramatically promoted by Fenton’s reagent (that generates hydroxyl free radical) and is suppressed by free radical scavengers, such as DTT, NAC, Vitamin C, and α-lipoid acid. UV radiation on cultured cells also promotes DSS1-protein association markedly. These observations suggest that DSS1 mainly targets oxidized proteins to form adducts. The authors also found that the formation of DSS1-protein adducts is enhanced by ATP supplementation in a dose-dependent manner and is blocked by the addition of metal chelator EDTA in the reaction mixture, suggesting that this process requires energy and involves ATPase. Additional experiments showing that the denatured cell lysate does not form DSS1-protein adducts further support that the DSS1 modification may be an enzyme-catalyzed process. By using liquid chromatography coupled with tandem mass spectrometry (LC-MS/MS), Zhang et al. identified 39 proteins from DSS1 conjugates affinity-purified from the lysates of the cells after exposure with UV radiation. These DSS1-modified proteins are involved in a wide range of important biological events and pathways. The wide diversity of proteins modified by DSSylation implies that this posttranslational modification might be a fundamental process in biology.

The nature of protein modification by DSS1 remains elusive. Initial evidence observed by Zhang et al. suggests a one-to-one interaction between DSS1 and the target protein. Because of this stoichiometry and the likely enzymatic reaction of the modification, it is likely that DSS1 conjugates with target proteins through a covalent bond. Mutation of one of the four conserved aromatic amino acid residues (W27, W39, W43 or F52) of DSS1 molecule into glycine or alanine leads to a marked decrease (~50%–70%) in its capacity to associate with cellular proteins. If three of these four residues are replaced, the mutant DSS1 almost becomes unable to bind to target proteins. Thus, these four residues of DSS1 are critical for the formation of the DSS1-protein adducts. It remains to be determined whether DDS1 conjugates with target proteins through the side-chain of tryptophan and what chemical bond bridges the binding.

Initial evidence reported by Zhang et al. indicates that DSSylation may mainly modify oxidized proteins and the DSSylated proteins are readily degraded through the ubiquitin-proteasome system. These observations hint a potentially important biological implication of protein DSSylation. As Zhang et al. proposed based on their findings, aberrantly oxidized proteins might first be marked by DSS1 through DSSylation, and then the DSSylated proteins are recognized by E3 ubiquitin ligases, leading to the degradation of the target proteins through the ubiquitin-proteasome system. This hypothesis is consistent with previous findings of DSS1’s involvement in protein degradation (Funakoshi et al., 2004; Sone et al., 2004; Wei et al., 2008) and with the fact that a loss of the *DSS1* gene results in an increased sensitivity to damage caused by oxidative stressors, such as chemicals, UV, and radiation (Kojic et al., 2003; Funakoshi et al., 2004). If this hypothesis is proved to be correct by independent studies and direct evidence in the future, this will be an important fundamental mechanism for removing proteins damaged by oxidative stress inside the cell. As DSS1 is highly conserved in all eukaryotic species, this mechanism might be an ancient protective response universally conserved in eukaryotes.

Under physiological conditions, any damaged, misfolded, and senescent proteins are efficiently cleared from inside the cell by protein degradation machineries involving the ubiquitin-proteosome system and lysosomes. If cells are under stress or the formation of damaged or misfolded proteins exceeds the capacity of protein degradation, the misfolded proteins aggregate inside the cells, which could eventually choke the cell to death. The same consequence may occur if the protein degradation machinery is deficient. Many diseases, collectively called proteopathies, are caused or involved in misfolded protein aggregation. The most common neurodegenerative diseases, such as Alzheimer’s disease and Parkinson’s disease, belong to this category.

Several mechanisms can result in protein misfolding (Menzies et al., 2011), but the exact mechanisms are less clear. Oxidative stress is one of the cellular insults promoting protein misfolding. Oxidative stress has been widely implicated in various physiological and pathological processes, including aging and many age-related diseases. It is believed to play an important role in the development of several neurodegenerative diseases, such as Alzheimer’s disease (Bonda et al., 2010) and Parkinson’s disease (Hauser and Hastings, 2012). One initiative factor for the pathogeneses of these diseases is oxidative damage, leading to accumulation of oxidized proteins inside of the affected neurons. It is intriguing to investigate if protein DSSylation is impaired under these pathological conditions. Understanding the DSSylation modification of protein will certainly help uncover the mysteries of these disorders and might also provide a novel target for developing the prevention and treatment of these disorders.

The study reported by Zhang et al. in this issue provide a set of initial evidence that suggests a potentially crucial mechanism for maintaining protein homeostasis and protecting cells from oxidative insults. As the implications of this possible mechanism are enormous both in biology and in pathological conditions, this study may open a whole new research field related to proteostasis, oxidative stress, and proteopathies. Like many other initial breakthrough studies, this one by Zhang et al. provides more questions than answers. What is the chemical nature of protein DSSylation? What enzyme(s) catalyze the binding of DSS1 to the target proteins? How does DSS1 sense proteins damaged by oxidative stress? How do E3 ubiquitin ligases recognize DSSylated proteins? Which and how many E3 ligases participate in this process? Is DSSylation reaction reversible? Is DSS1 recycled after the target protein is ubiquitinated? Is DSSylation protective or deleterious or both depending on the individual situations? Does DSS1 also modify other oxidized large molecules like DNA and RNA? Answering these questions will take many ambitious scientists many years.

DSS1 is previously known to be involved in many important biological functions and cellular processes, such as genome stability (Marston et al., 1999), homologous recombination and DNA repair (Yang et al., 2002), cellular proliferation and neoplastic transformation (Wei et al., 2003), protein degradation (Funakoshi et al., 2004; Sone et al., 2004; Wei et al., 2008), histone modification (Qin et al., 2009), and mRNA splicing, metabolism, and export (Thakurta et al., 2005; Wilmes et al., 2008). The study described by Zhang et al. in this issue demonstrates a novel role of DSS1 protein and a novel type of posttranslational protein modification targeting oxidized proteins for degradation through the ubiquitin-proteasome system. If this study can stand the test of time, it will be a milestone discovery in biology.

## References

[CR1] Bonda DJ, Wang X, Perry G, Nunomura A, Tabaton M, Zhu X, Smith MA (2010). Oxidative stress in Alzheimer disease: a possibility for prevention. Neuropharmacology.

[CR2] Crackower MA, Scherer SW, Rommens JM, Hui C-C, Poorkaj P, Soder S, Cobben JM, Hudgins L, Evans JP, Tsui L-C (1996). Characterization of the split hand/split foot malformation locus SHFM1 at 7q21. 3-q22. 1 and analysis of a candidate gene for its expression during limb development. Hum Mol Genet.

[CR3] Funakoshi M, Li X, Velichutina I, Hochstrasser M, Kobayashi H (2004). Sem1, the yeast ortholog of a human BRCA2-binding protein, is a component of the proteasome regulatory particle that enhances proteasome stability. J Cell Sci.

[CR4] Hauser DN, Hastings TG (2012). Mitochondrial dysfunction and oxidative stress in Parkinson’s disease and monogenic parkinsonism. Neurobiol Dis.

[CR5] Kojic M, Yang H, Kostrub CF, Pavletich NP, Holloman WK (2003). The BRCA2-interacting protein DSS1 is vital for DNA repair, recombination, and genome stability in *Ustilago maydis*. Mol Cell.

[CR6] Marston NJ, Richards WJ, Hughes D, Bertwistle D, Marshall CJ, Ashworth A (1999). Interaction between the product of the breast cancer susceptibility gene BRCA2 and DSS1, a protein functionally conserved from yeast to mammals. Mol Cell Biol.

[CR7] Menzies FM, Moreau K, Rubinsztein DC (2011). Protein misfolding disorders and macroautophagy. Curr Opin Cell Biol.

[CR8] Qin S, Wang Q, Ray A, Wani G, Zhao Q, Bhaumik SR, Wani AA (2009). Sem1p and Ubp6p orchestrate telomeric silencing by modulating histone H2B ubiquitination and H3 acetylation. Nucleic Acids Res.

[CR9] Sone T, Saeki Y, Toh-e A, Yokosawa H (2004). Sem1p is a novel subunit of the 26 S proteasome from *Saccharomyces cerevisiae*. J Biol Chem.

[CR10] Thakurta AG, Gopal G, Yoon JH, Kozak L, Dhar R (2005). Homolog of BRCA2-interacting Dss1p and Uap56p link Mlo3p and Rae1p for mRNA export in fission yeast. EMBO J.

[CR11] Wei S-J, Trempus CS, Cannon RE, Bortner CD, Tennant RW (2003). Identification of Dss1 as a 12-O-tetradecanoylphorbol-13-acetate-responsive gene expressed in keratinocyte progenitor cells, with possible involvement in early skin tumorigenesis. J Biol Chem.

[CR12] Wei S-J, Williams JG, Dang H, Darden TA, Betz BL, Humble MM, Chang F-M, Trempus CS, Johnson K, Cannon RE (2008). Identification of a specific motif of the DSS1 protein required for proteasome interaction and p53 protein degradation. J Mol Biol.

[CR13] Wilmes GM, Bergkessel M, Bandyopadhyay S, Shales M, Braberg H, Cagney G, Collins SR, Whitworth GB, Kress TL, Weissman JS (2008). A genetic interaction map of RNA-processing factors reveals links between Sem1/Dss1-containing complexes and mRNA export and splicing. Mol Cell.

[CR14] Yang H, Jeffrey PD, Miller J, Kinnucan E, Sun Y, Thomä NH, Zheng N, Chen P-L, Lee W-H, Pavletich NP (2002). BRCA2 function in DNA binding and recombination from a BRCA2-DSS1-ssDNA structure. Science.

[CR15] Zhang Y, Chang F-M, Huang J, Junco JJ, Maffi SK, Pridgen HI, Catano G, Dang H, Ding X, Yang F, Kim DJ, Slaga TJ, He R, Wei S-J (2014) DSSylation, a novel protein modification targets proteins induced by oxidative stress, and facilitates their degradation in cells. Prot Cell. doi:10.1007/s13238-013-0018-8 10.1007/s13238-013-0018-8 PMC3956975 24515614

